# AXL as a Target in Breast Cancer Therapy

**DOI:** 10.1155/2020/5291952

**Published:** 2020-02-14

**Authors:** Sierra A. Colavito

**Affiliations:** Department of Biology, University of Wisconsin-La Crosse, La Crosse, WI 54601, USA

## Abstract

AXL is a receptor tyrosine kinase (RTK) that has been implicated in diverse tumor-promoting processes such as proliferation, migration, invasion, survival, and apoptosis. AXL therefore plays a role in cancer progression, and AXL has been implicated in a wide variety of malignancies from solid tumors to hematopoietic cancers where it is often associated with poor prognosis. In cancer, AXL has been shown to promote epithelial to mesenchymal transition (EMT), metastasis formation, drug resistance, and a role for AXL in modulation of the tumor microenvironment and immune response has been identified. In light of these activities multiple AXL inhibitors have been developed, and several of these have entered clinical trials in the U.S. In breast cancer, high levels of AXL expression have been observed. The role of AXL in cancer with a focus on therapeutic implications for breast cancer is discussed.

## 1. Introduction

AXL is an RTK that is part of the TAM (TYRO3, AXL, and MER) family of RTKs. AXL was originally identified in 1988 during a screen for genes involved in the progression of chronic myelogenous leukemia (CML) to blast crisis [1]. Approximately three years after AXL's initial discovery, two groups independently cloned and identified AXL as an RTK with transforming potential [2, 3]. While AXL was found to be necessary for transformation, it alone was not sufficient [3]. Initially, the intracellular role of AXL remained a mystery, as evidenced by one of these groups giving AXL the name “UFO” in reference to its unknown function [2]. Since then, AXL has been shown to be involved in a variety of cellular processes, including cell growth, proliferation, survival, apoptosis, and adhesion. Given these functions of AXL, it is not surprising that AXL plays a role in cancer progression, and indeed AXL has been implicated in a wide variety of malignancies from solid to liquid tumors. In breast cancer AXL expression has been observed in all of the main transcriptional subtypes, and AXL expression in primary breast tumors is strongly predictive of reduced patient survival and poor outcome [4, 5].

## 2. AXL Signaling Axis

**Figure 1:**
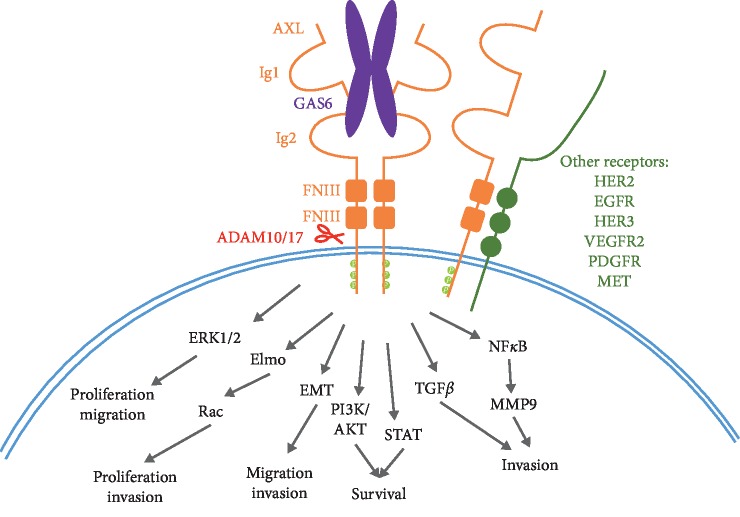
In breast cancer, AXL (orange) can be activated through either binding of GAS6 (purple) or through interaction with other receptors (green) to activate a variety of downstream signaling pathways (gray). Cleavage by ADAM10/17 (red) can result in release of the extracellular domain which retains ligand-binding abilities.

Structurally, AXL, like the other TAM family members, consists of two immunoglobulin- (Ig-) like domains and two fibronectin III domains which comprise the extracellular portion of the receptor (Figure 1) [6]. It is through the fibronectin domains that AXL is thought to exert its effects on adhesion, which relates to such cellular processes as EMT, whereby polarized epithelial cells undergo a shift to a more mesenchymal-like state. The intracellular portion of AXL consists of a receptor tyrosine kinase domain containing a KWIAIES motif that is conserved among the TAM family members, though in TYRO3 the isoleucines are substituted with leucine residues [3, 7].

Canonical AXL activation involves binding of the ligand growth arrest-specific 6 (GAS6) to the Ig-like domains on AXL, resulting in receptor dimerization. GAS6 is able to bind the other TAM family receptors, but it shows a much higher affinity for AXL [8]. Up until recently protein S was thought to exclusively be a ligand for TYRO3 and MER, but recently it has been shown to be capable of binding to and activating AXL in glioblastoma cells [9]. Activation of AXL is not complete until a further interaction with the phospholipid phosphatidyl serine (PS) occurs, mediated by the gamma-carboxyglutamic acid (Gla) domain on GAS6 following its posttranslational modification [10]. PS is a phospholipid that is normally restricted to the intracellular portion of the phospholipid bilayer but is externalized in apoptotic cells or cells that are otherwise stressed, such as in virally infected cells. The tumor microenvironment also contains a high level of externalized PS due to the increased apoptotic index of tumors, metabolically stressed tumor cells, vasculature within the tumor, and tumor-derived exosomes [11].

Activation of AXL results in autophosphorylation on tyrosine residues in the cytoplasmic domain of the receptor and subsequent phosphorylation and activation of adaptor signaling proteins resulting in a signaling cascade and phosphorylation of downstream targets. The phosphorylation sites on AXL and activation of downstream pathways are highly context-dependent. Multiple tyrosine phosphorylation sites have been identified in the intracellular domain of AXL: Y698, Y702, Y703, Y779, Y821, and Y866, and three of these have been shown to be phosphorylated in breast cancer or breast cancer cell lines: Y698, Y702, and Y703 [12–15]. These three tyrosines represent sites of autophosphorylation and thus activation of AXL, with the remaining residues thought to be involved in signaling and docking of adaptor proteins.

## 3. Alternative Methods of Activation

Ligand-independent activation of AXL has also been observed. In MCF-7 cells for instance, activation of AXL independently of GAS6 binding stimulated nuclear factor-kappa B (NF-*κ*B) mediated activation of MMP-9, although the exact mechanism by which AXL was activated in this case was not determined [16]. Similarly, in breast cancer stem cells GAS6 did not have an effect on AXL activation status, and while AXL was expressed at high levels in these cells GAS6 transcripts were only found at low levels, indicating that AXL activation in breast cancer stem cells is likely GAS6-independent [17].

AXL can be activated by interaction with other receptors or membrane-bound molecules, including by itself. Ectopic overexpression of AXL can result in receptor homodimerization and activation in cell lines such as NIH 3T3 cells; however, it is not known if this occurs in an *in vivo* setting [18].

AXL has been shown to interact with EGFR (epidermal growth factor receptor) and other EGFR family members. In triple-negative breast cancer (TNBC) lines AXL can be activated by EGFR [19]. This activation by EGFR expands the downstream signaling pathways beyond those seen when EGFR is activated alone. AXL can also promote the translocation of EGFR to the nucleus [20]. Meyer et al. also observed an interaction between AXL and other EGFR family members, namely HER2 and HER3 in breast cancer cells, as well as an interaction between AXL and MET (hepatocyte growth factor receptor) and AXL and PDGFR (platelet-derived growth factor receptor). Interactions between AXL and these other RTKs lead to enhanced downstream signaling. An interaction between AXL and EGFR has also been observed in glioblastoma cell lines where EGF can activate AXL, and the interaction between EGFR and AXL can lead to MMP9 activation to promote invasion [21].

The effect of the interaction between AXL and HER2 in breast cancer has recently been clarified. AXL can interact with HER2, both when HER2 is ectopically overexpressed in MCF10a cells and endogenously in the HER2-amplified SKBR3 cell line [4]. This interaction leads to increased levels of AXL localized to the cell membrane and HER2 transphosphorylation of AXL, although AXL is not able to phosphorylate HER2. This interaction enhanced invasiveness and other functions important for metastasis formation. Importantly, this interaction between AXL and HER2 was also observed in HER2^+^ patient tumors [4].

Additionally, reverse-phase protein array analyses in breast tumors revealed evidence of cross-talk between AXL and MET, along with potential physical interaction between these two receptors [22]. In neuronal cells, AXL exhibits cross-talk with MET, whereby regulation of neuronal survival by AXL is reliant on MET, not GAS6 [23].

Other means of ligand-independent activation of AXL have also been reported in other cell types through proteins such as vascular endothelial growth factor receptor-2 (VEGFR-2) and C1-TEN, although these have not been analyzed in breast cancer cells [24, 25]. Activation of AXL through interaction with other RTKs suggests that combination therapies that target both AXL and its interacting partners may be necessary in the clinical setting.

## 4. Post-Transcriptional and Post-Translational Modifications

Three alternative splicing variants have been identified for AXL, two of which encode 4.7 kb long mRNAs which only differ by 27 basepairs which are internal in the mRNA. Therefore, both of these isoforms contain intact extracellular ligand-binding domains and intracellular kinase domains, and both can effect cellular transformation [3]. The third isoform is the shortest, owing to exclusion of five alternative exons in the 5′ coding region of the mRNA. It is not clear if there are specific differences in the function of these isoforms in cancer.

Post-translational modification of AXL can also alter AXL's role in the cell. The phosphorylation of AXL which occurs following its activation targets AXL for ubiquitinated-mediated degradation [26, 27]. An alternative method of degradation is proteolytic cleavage by matrix metalloproteinases and A Disintegrin and Metalloproteinase Domain (ADAM) family members in the extracellular domain, which releases a soluble 85 kDa N-terminal fragment of AXL (sAXL) [28]. The extracellular domain contains intact ligand-binding capabilities, and therefore sAXL can inhibit ligand-mediated AXL signaling [29]. sAXL levels are elevated in some cancer types, including hepatocellular carcinoma, and sAXL is being investigated as a biomarker in certain cancers and other inflammatory diseases [30–32].

AXL is post-translationally modified by glycosylation in the golgi. The extracellular domain of AXL contains six N-linked glycosylation sites. Fully glycosylated AXL is 140 kDa, with partial glycosylation yielding a protein of approximately 120 kDa. Inhibition of N-acetylglucosamine (GlcNAc) transferase results in accumulation of a 100 kDa unglycosylated form of AXL [33]. It is thought that glycosylation is important for AXL's function, as inhibition of glycosylation results in a decrease in cell proliferation and invasion in hepatocellular carcinoma cell lines and in metastasis formation *in vivo* [33].

## 5. Downstream Signaling Targets and Effectors in Breast Cancer

Like that of most RTKs AXL can signal through a variety of downstream effectors leading to influences on a variety of intracellular processes that are highly context-dependent. In breast cancer, AXL can stimulate a variety of downstream pathways, including phosphatidylinositide 3-kinase/RAC-*α* serine/threonine protein kinase (PI3K/AKT), extracellular signal-regulated kinase (ERK), p38 mitogen-activated protein kinase (MAPK), and the NF-*κ*B pathway, as well as signal transducer and activator of transcription signaling (STAT) pathways [34]. Activation of these pathways in breast cancer can stimulate a variety of tumor-promoting abilities including breast cancer cell motility, invasion, proliferation, survival, angiogenesis, and other processes (Figure 1).

### 5.1. GAS6 Independent Pathways

In AXL^–/–^/HER2^+^ mouse tumor models, a decrease in EGF, Rho-GTPase, and TGF-*β* (transforming growth factor *β*) signaling is observed compared to in AXL-competent cells, indicating that AXL promotes signaling through these pathways [4]. These downstream effectors affect cellular processes such as extracellular matrix organization, cell migration, cytoskeletal organization, and EMT [4]. Stimulation of AXL and these subsequent downstream pathways appears to be ligand-independent in HER2^+^ breast cancer [4].

In MCF-7 cell lines as well as in inflammatory breast cancer, AXL can promote cell invasion by inducing MMP-9 through NF-*κ*B independently of GAS6 binding [16, 35]. AXL activation via interaction with EGFR amplifies signaling through the AKT pathway and elaborates on downstream EGFR signaling to include downstream pathways such as GSK3 (glycogen synthase kinase 3), ERK, and P38 in TNBC cells [19]. These pathways affect cellular processes such as proliferation and migration.

Reverse-phase protein array analysis in breast tumors revealed evidence of cross-talk between AXL and the RTK MET, along with potential physical interaction between these two receptors [22]. Treatment of MDA-MB-231 cells with the MET ligand HGF resulted in phosphorylation of AXL, and knockdown of MET reduced AXL mRNA levels [22]. Although the two receptors could coimmunoprecipitate this is not entirely a ligand-independent interaction, as MET was observed to be required for signal transduction by GAS6 [22]. The downstream effectors of this interaction include AKT and ERK and are thought to be important for apoptosis and migratory ability in MDA-MB-231 cells.

### 5.2. Ligand-Mediated Downstream Signaling Pathways

Several different downstream pathways can be activated through ligand-dependent activation of AXL. A reverse-phase protein analysis of human breast tumors indicated that GAS6 can promote phosphorylation of several downstream targets including AKT, CREB, GSK3*α*/*β*, ERK1, c-Jun, MEK1, S6, Stat3, as well as NF*κ*B [22]. Similarly, stimulation of MDA-MB-231 cells with GAS6 resulted in activation of various downstream signaling cascades including PI3K-AKT, MAP kinase, NF-*κ*B, and JAK-STAT pathways [36].

Immunoprecipitation of AXL from lapatinib-resistant HER2^+^ cells contained a subunit of PI3K, indicating that AXL and PI3K may have a functional interaction [37]. Additionally, knockdown of AXL in MDA-MB-231 cells leads to a decrease in proangiogenic factors released into the media, including VEGF, thrombospondin-1, endothelin-1, and uPA [38]. Therefore, AXL could also mediate effects on the recruitment of blood vessels to the solid breast tumor.

Another means by which AXL can promote invasion is through GAS6-mediated phosphorylation of the Elmo scaffold proteins by AXL to ultimately activate Rac GTPase, which also promotes breast cancer cell proliferation [39]. AXL activation through binding to macrophage-produced GAS6 can lead to downstream activation of AKT and STAT3, promoting survival in a p53 ^–/–^ model of early-stage mammary tumors [40].

## 6. Regulation of AXL Expression in Breast Cancer

Despite the elevated levels of AXL seen in breast malignancies, less than 2.5% of breast cancers exhibit any alteration in *AXL* (mutation, rearrangement, etc.), and amplifications specifically are also exceedingly rare (less than 2%) [41–43]. Therefore, it is likely that the contribution of AXL to transformation is likely due to overexpression of the wild-type receptor, which can be mediated through various mechanisms.

Several transcription factors have been found to directly regulate AXL expression in breast cancer. A ChIP-seq analysis in MDA-MB-231 cells identified AXL as a target of the Hippo transducers YAP/TAZ [44]. This confirmed earlier reports in other cancer types that AXL is regulated by YAP/TAZ [45, 46]. Vimentin and other EMT transcription factors can also regulate AXL, including Twist, Snail, and Slug [5, 47].

Other transcription factors have been implicated in the expression of AXL in other cancer types, but these have not been directly studied in breast cancer. The transcription factor HIF*α* can promote AXL in renal clear cell carcinoma, and AP1 can promote AXL expression in leukemia [48, 49]. The AP1 transcription factors c-JUN and c-FOS can mediate AXL overexpression in head and neck and esophageal cancers [50]. Exogenous expression of the Sp1 and Sp3 transcription factors can increase AXL transcripts in several cancer cell lines, although this has not been examined in breast cancer [51]. Additionally, MZF1 binds to the AXL promoter in cervical and colorectal cancer cell lines and enhances transcription of AXL [52].

In addition to regulation by transcription factors, several other players have been shown to affect AXL transcript levels in breast cancer cells. Knockdown of the MET RTK results in a reduction of AXL mRNA levels in MDA-MB-231 cells [22]. A systems modeling approach identified decreased AXL expression following MEK inhibition in TNBC cells [53]. AXL expression has been shown to correlate with that of the estrogen (ER) and progesterone (PR) receptors in breast cancer, indicating that estrogen and/or progesterone could regulate AXL expression [54, 55]. Indeed, blocking estrogen through either use of an estrogen antagonist or estrogen deprivation results in lower AXL mRNA levels in HER2^+^ ER^+^ breast cancer cells that have acquired resistance to lapatinib [37].

Epigenetic modifications such as promoter methylation and histone acetylation can also affect AXL transcript levels. Decreased methylation of the AXL promoter was observed in lapatinib-resistant HER2^+^ breast cancer cells compared to sensitive cells, which correlated with higher levels of AXL expression in the resistant cells [37]. In colorectal and cervical cancer cell lines, methylation of certain transcription factor binding sites in the AXL promoter lead to decreased expression of AXL [51]. In lung cancer, mutant p53 bound to the AXL promoter and could reduce histone acetylation, leading to upregulation of AXL expression and enhanced cell growth and motility [56]. And finally, in acute myeloid leukemia (AML), methylation of the AXL promoter was inversely correlated with response to chemotherapy drugs [57].

MicroRNAs have also been implicated in regulation of AXL post-transcriptionally. miR-34a targets the *AXL* 3′ UTR in TNBC (MDA-MB-231 cell line). mIR-34a is found at a low level in MDA-MB-231 cells compared to nontransformed MCF10a cells or other lines representing other breast cancer subtypes [58, 59]. When miR-34a was overexpressed, AXL was downregulated [58]. Other microRNAs can regulate AXL in other cancer types, including miR-199a-3p in osteosarcoma and miR-139 in prostate cancer [60–62].

AXL can also be regulated at the protein level. The membrane protein TIG1 binds and stabilizes AXL in breast cancer, preventing its proteasomal degradation leading to enhanced proliferative, migratory, and invasive abilities via activation of NF-*κ*B and MMP-9 [35]. As mentioned above, protease cleavage can lead to release of the cytoplasmic domain of the receptor, which retains ligand-binding capabilities. Therefore, regulation of cleavage could alter AXL activity levels.

## 7. The Role of AXL in the Tumor

Up until recently, AXL expression was thought to be limited to TNBC cell lines. However, analysis of patient tumors indicates that AXL is present across all of the main transcriptional subtypes, and there is no significant association between AXL expression and TNBC [4, 63]. Additionally, others have shown that AXL expression correlates with the presence of the estrogen receptor in breast carcinomas, indicating that despite high-level expression of AXL in TNBC, AXL is not restricted to TNBC tumors [54]. Indeed, a role for AXL in the progression of various breast cancer subtypes has been identified. These include in inflammatory breast cancer [35], TNBC [64], and ER-positive [54], as well as HER2-amplified [4] breast cancers. AXL has also been implicated in more rare forms of breast tumors, including in phyllodes tumors of the breast, which are tumors that originate in stromal tissue [65], as well as in male breast cancer [66]. The role of AXL in the processes of EMT, metastasis, and the microenvironment/immune system in breast cancer is discussed below.

## 8. EMT

EMT describes the process whereby cells undergo a morphological transition from the epithelial polarized phenotype to the mesenchymal fibroblastoid phenotype. This process is defined by loss of cell-cell adhesion molecules, such as E-cadherin, downregulation of epithelial differentiation markers, and upregulation of mesenchymal molecular markers, such as vimentin. This process is crucial for normal embryonic development, and it also plays a role in tissue regeneration, organ fibrosis, inflammation, and tumor progression. In cancer, it is hypothesized that EMT cells gain migratory, invasive, and survival potential at the expense of proliferative ability. EMT has therefore been implicated in the process of metastasis. AXL expression strongly correlates with EMT markers across a spectrum of cancer types, including breast, lung, colorectal, bladder, endometrial, and ovarian cancers [67].

Originally, AXL was identified as a downstream target of EMT in breast cancer, whereby vimentin was shown to upregulate Slug and ultimately AXL [47]. Similarly, induction of EMT in the nontumorigenic epithelial MCF10a cell line through overexpression of the EMT transcription factors Snail, Slug, Twist, or Zeb2 resulted in elevated levels of AXL expression [5]. AXL is expressed at higher levels in mesenchymal mammary cell lines compared to parental epithelial lines, and if the mesenchymal cells are induced to become more epithelial like through a mesenchymal to epithelial transition, then these cells have less AXL expression [17]. Induction of EMT in MCF10a cells was also observed to promote autocrine GAS6 signaling [5]. These studies indicate that AXL is downstream of EMT.

However, more recently AXL has been identified as also being able to promote EMT, and therefore functions as part of a positive feedback loop to promote EMT as well as be upregulated in response to EMT transcription factors in normal and immortalized mammary cells [17]. Overexpression of AXL in human mammary epithelial cells (HMLE) resulted in a decrease in E-cadherin and an increase in the mesenchymal markers N-cadherin, vimentin, and Snail, consistent with an EMT [17]. Additionally, treatment of TNBC cell lines with recombinant GAS6 results in increased expression of EMT-associated genes, such as *SNAIL*, *SLUG*, and *VIM*, which effected increased migration and invasion [68].

siRNA knockdown of vimentin leads to a decrease in AXL expression in MCF10a cells, which are immortalized mammary cells but are not transformed [47]. A correlation was seen between the mesenchymal marker vimentin and AXL in both migratory cells at the wound edge in cell-based assays as well as in mammary tumors [47]. Interestingly, AXL and vimentin expression also correlated in normal mammary tissue samples from patients, not just in tumor tissue [47]. When vimentin is targeted by siRNAs in the mesenchymal MDA-MB-231 cell line, AXL protein levels decreased by 56%; however, no change in vimentin levels was observed when AXL was targeted [47].

AXL does not just play a role in mesenchymal cancers. Neu^+^: *AXL*^–*/*–^ tumors grown in mice had decreased expression of EMT genes compared to those with wild-type AXL expression and AXL expression in HER2^+^ tumors correlated with EMT markers [4]. HER2^+^ cancers retain epithelial features, but there is evidence that their metastasis requires EMT [69].

## 9. Metastasis

AXL has been shown to promote breast cancer cell motility, invasion, proliferation, survival, and anoikis resistance, often in conjunction with its role in EMT. Not surprisingly then AXL has been implicated in breast cancer metastasis. AXL levels have been observed to be increased in metastases compared to the primary tumor of the same patient [5].

Overexpression of AXL can induce increased invasiveness and motility of the normally weakly invasive MCF-7 cell line [70], and AXL knockdown in the mesenchymal/invasive MDA-MB-231 cell line results in decreased migratory and invasive ability [5, 47, 71].

AXL expression is also associated with increased tumorigenecity of breast cancer cells. In mouse breast cancer cells sorted into two groups based on the level of AXL expression, it was observed that cells expressing high levels of AXL were more tumorigenic than their low AXL-expressing counterparts [17]. In a mouse model of HER2^+^ breast cancer, only slight differences were observed in tumor formation in AXL knockout mice compared to controls [4]. This is in contrast to tumor formation by MDA-MB-231 cells, a mesenchymal triple-negative cell line, where AXL was shown to be required for primary tumor formation, and knocking down AXL expression in established tumors reduces their size [5, 71]. In a mouse model of radioresistant breast cancer, MMTV-PyMT tumors selected for resistance to radiation therapy, AXL knockout reduced tumor growth compared to control tumors [72].

A direct role for AXL in promoting breast cancer metastasis has also been observed. In an *in vivo* comparison between metastasis formation of MDA-MB-231 cells with and without AXL knockdown, control cells metastasized to the lymph nodes, lungs, ovaries, and kidneys, while no macro- or micrometastases were observed from the AXL knockdown cells [5]. A similar experiment looked for metastasis specifically to the lung. While AXL knockdown in MDA-MB-231 xenografts was able to form tumors, there were no observable metastases to the lungs in AXL knockdown tumors [71].

Despite not showing a significant effect on primary tumor formation, in a model of HER2^+^ breast cancer when metastasis formation was tracked, AXL knockout mice showed reduced lung metastases compared to controls [4]. GAS6 knockout did not affect metastasis formation in these same studies, indicating that the metastasis-promoting effect of AXL occurs independently of GAS6 and is thought to involve interaction between AXL and HER2 [4]. AXL was shown to be important for all steps of the metastatic process in this model. By analyzing the amount of circulating tumor cells in orthotopic xenografts derived from HER2^+^ control and AXL knockout cells, researchers observed a reduced number of circulating cells derived from xenografts in the AXL knockout cell lines compared to controls, indicating that AXL is important for initial intravasation of the primary tumor [4]. A similar decrease in extravasation was seen with AXL knockdown, and AXL was shown to be required for the maintenance of HER2^+^ metastatic lesions as induction of shRNAs targeting AXL in the period after extravasation into the lungs had occurred resulted in smaller metastases [4].

The role of AXL in metastasis of other subtypes of breast cancer is less clear. When injected into the tail vein of mice, MDA-MB-231 cells with AXL knockdown extravasated into the lungs significantly less than for cells without AXL knockdown, indicating that AXL is important for extravasation [47]. However, AXL was shown to be dispensable for the maintenance of already established lung metastases from MDA-MB-231 xenografts, where induction of AXL knockdown after lung colonization had occurred resulted in the same amount of lung foci compared to controls without AXL knockdown [71]. In a model of TNBC breast cancer, xenografts of MDA-MB-231 cells showed reduced tumor growth with AXL knockdown [5]. AXL was also observed to be essential for metastasis of these tumors as well as metastasis from the highly metastatic mouse 4T1 tumor model [5]. This aligns with other evidence cited above that AXL is primarily responsible for metastasis formation and not primary tumor growth in epithelial-like cancers (ER^+^, HER2^+^), while it may play a role in primary tumor formation (and metastasis) if the primary tumor is mesenchymal-like, as is the case for most TNBC cancers.

AXL has therefore been implicated in intravasation, extravasation, and maintenance of metastases in HER2^+^ breast cancer and in extravasation in other models of mammary tumors. In HER2^+^ cancers at least, GAS6 appears to be dispensable for metastasis [4]. This does not mean GAS6 is uninvolved in metastasis as it can play an important role in reprogramming of the metastatic niche to promote metastasis outgrowth, discussed below.

## 10. Microenvironment and Immune System

A role for GAS6 has also been observed in modulating the tumor microenvironment. In a mouse p53^–/–^ model of breast cancer, increased GAS6 levels were observed in preinvasive lesions that have high levels of infiltrating macrophages [40]. These immune system macrophages can produce GAS6, stimulating AXL and leading to activation of downstream pathways AKT and STAT3, resulting ultimately in decreased E-cadherin expression [40]. Increased GAS6 in preinvasive hyperplastic lesions compared to the normal mammary gland was observed, but decreased GAS6 levels were seen in invasive tumors compared to preinvasive lesions, even though macrophages continued to increase. In GAS6^–/–^ knockout mice, early stage progression and time to tumor formation are decreased, but established tumor growth is unaffected [40]. In sum these data suggest that stromal GAS6 is involved in early changes that promote the switch from preinvasive to invasive cancer.

In a study of mouse 4T1 cells, which are highly metastatic breast cancer cells, leukocyte-derived GAS6 was shown to promote tumor growth *in vivo* [73]. In GAS6^–/–^ mice, 4T1 tumors can grow, but they are smaller than those grown in GAS6^+/+^ mice, likely owing to decreased proliferation, not increased apoptosis in these tumors. In GAS6^–/–^ mice, metastasis to the lungs is also reduced but not eliminated [73].

A direct role for AXL in modulation of the immune system has also been observed. In the MMTV-PyMT mouse model of breast cancer, AXL was observed to be upregulated in mesenchymal extravasated cells and to be important for activating lung fibroblasts in the stroma. These activated fibroblasts in turn secrete factors which favor the epithelial state, resulting ultimately in a mesenchymal to epithelial transition (MET) that promotes metastasis growth [74]. Thus, AXL promotes reprogramming of the metastatic niche that favors both an initial EMT and a subsequent MET at the metastatic site.

High-level expression of AXL in mouse mammary tumors that were unresponsive to ionizing radiation in combination with immune checkpoint therapy has also been observed [72]. When AXL was deleted from these resistant tumors they became radiosensitive, which was mediated by immunological alteration, including enhanced antigen presentation and altered cytokine secretion [72]. AXL can suppress MHCI expression and thus antigen presentation and promote the release of cytokines that further contribute to decreasing the antitumor immune response.

In other cancer types, AXL can upregulate immune checkpoint proteins and alter chemokine signaling in lung adenocarcinoma, suggesting therapies that target immune checkpoint molecules like anti-PDL1 might be ineffective unless a combination therapy is used which also targets AXL [75]. Given the role of AXL in altering the immune system in breast cancer, a similar mechanism could be occurring in mammary tumorigenesis as well.

## 11. Clinical Implications

The above-discussed roles of AXL in the cell and tumor result in clinical implications for AXL in breast cancer. AXL and its ligand GAS6 could have prognostic and/or biomarker potential in the clinic. AXL is a strong negative predictor of patient survival in breast cancer, indicating its prognostic potential [5]. Similarly, AXL-associated tumor inflammation is correlated with poor prognosis in TNBC patients [76]. Additionally, levels of GAS6 could also be of prognostic significance in the clinical setting. Macrophage-derived GAS6 is a critical regulator of the transition from premalignant to invasive cancer and could be a biomarker of progression for patients with early-stage cancer [40].

sAXL, the cleaved extracellular domain of AXL, is being explored as a potential biomarker in certain cancers and other inflammatory conditions, such as in hepatocellular carcinoma, neurofibromatosis type 1, and in NSCLC [30–32, 77]. sAXL was overexpressed in effusions from patients with breast carcinoma; however, this was not informative of chemoresponse or survival [78]. Therefore, it is currently unclear whether sAXL levels could be used as a biomarker in breast cancer.

## 12. Resistance to Therapy

AXL expression in the tumor has been implicated in resistance to a variety of therapies, both targeted and conventional, in several different cancer types including breast. Additionally, AXL expression in the microenvironment can contribute to drug resistance and epithelial plasticity [79].

### 12.1. Targeted Therapies

AXL has been implicated in resistance to EGFR-targeted therapy such as erlotinib and lapatinib in TNBC. This resistance is associated with EGFR-induced ligand-independent transactivation of AXL [19]. Activation of AXL has also been implicated in EGFR inhibitor resistance in HER2^+^ breast cancer [37]. AXL expression is upregulated in HER2^+^ cell lines with acquired resistance to lapatinib, a dual HER2 and EGFR inhibitor [37]. siRNA-mediated knockdown of AXL in these resistant cells restored sensitivity to lapatinib and to trastuzumab, a monoclonal antibody directed against HER2 [37].

AXL's ability to mediate resistance to EGFR-targeted therapies has been well studied in NSCLC, where mutation of EGFR is often a driver, and resistance to EGFR-targeted inhibitors is a major clinical hurdle. Treatment of NSCLC lines with an AXL inhibitor in combination with erlotinib restored sensitivity to erlotinib [80]. AXL is also able to mediate resistance to a newer EGFR-targeted inhibitor, osimertinib, in NSCLC [81–83]. This resistance is thought to be mediated by AXL interacting with EGFR and HER3 [82]. Finally, AXL has also been implicated in resistance to cetuximab treatment, a monoclonal EGFR-targeted antibody, in head and neck squamous cell carcinoma and in NSCLC [84].

In HER2-amplified gastric cancer cells, AXL, along with MET, can mediate resistance to afatinib, a pan-HER inhibitor [85]. It is not known if a similar mechanism is at play in HER2-amplifed breast cancer.

MEK inhibition in breast cancer is associated with decreased cleavage of the extracellular domain of AXL by ADAM10/17, resulting in increased signaling through AXL. This can confer resistance to MEK inhibitors and indicates a possible point of regulation of AXL activity in these cells [28].

Additionally, AXL has been implicated in resistance to a variety of other targeted therapies in other cancer types, including vemurafenib, sunitinib, alpelisib, crizotinib, and imatinib [86–90]. Resistance to such diverse therapies is likely linked to AXL's role in mediating EMT, which has long been associated with chemoresistance.

### 12.2. Conventional Therapies

AXL can also promote resistance to nontargeted agents. AXL-depleted murine breast cancer cells with breast cancer stem-cell-like properties were more sensitive to paclitaxel and etoposide compared to AXL-competent cells [17]. Similarly, inhibition of AXL signaling in MCF-7 cells with acquired resistance to conventional chemotherapies restored their sensitivity [91]. In TNBC and NSCLC cells that have undergone an EMT, inhibition of AXL can synergize with antimitotic agents such as docetaxel and paclitaxel but not with gemcitabine, doxorubicin, or cisplatin [92]. AXL inhibition in combination with other antimitotic agents, such as aurora kinase inhibitors and polo kinase inhibitors, which result in mitotic arrest, similarly resulted in synergistic inhibition of mesenchymal cancer cell proliferation [92]. As discussed above, AXL has been shown to promote EMT, and acquisition of mesenchymal features has been associated with resistance to a variety of targeted and conventional therapies [92].

In other cancer types, AXL has been implicated in resistance to doxorubicin and cytosine arabinoside in AML [57], to cisplatin resistance in esophageal cancer [93], to multiple chemotherapies in NSCLC [94], and to 5-FU treatment in colorectal cancer [95].

## 13. Inhibitors and Clinical Trials

Several inhibitors of AXL signaling have been investigated both in *in vitro* settings and in *in vivo* preclinical models and also in clinical trials. The mechanism of action of these therapeutics is diverse, including small molecular inhibitors, monoclonal antibodies, and CAR T-based therapies. Agents that have been used in clinical trials as well as some newer promising agents in preclinical development are discussed as follows.

## 14. Small Molecule Inhibitors

Several selective AXL or dual AXL/MER inhibitors have entered clinical trials; in addition, there are other multitarget AXL inhibitors that have been developed which target AXL in conjunction with other RTKs such as MET [96, 97].

### 14.1. AXL-Selective Inhibitors

#### 14.1.1. BGB324/Bemcentinib/R428

BGB324 is a small molecule AXL inhibitor that has entered clinical trials (Table 1). In preclinical models, BGB324 reduces the invasion of MDA-MB-231 and murine 4T1 cells, both of which are highly migratory and invasive cell lines [98]. In orthotopic models with 4T1 cells and intracardiac injection of MDA-MB-231 cells, BGB324 reduced the amount of metastases observed [98]. BGB324 alone or in combination with nivolumab, an anti-PD-1 antibody, prolonged the survival of mice with mesenchymal glioblastoma tumors [9]. Not just mesenchymal tumors have shown effects with BGB324 treatment. Long-term systemic treatment with BGB324 decreased circulating tumor cells and lung metastases in a mouse model of HER2^+^ breast cancer but had no effect on primary tumor growth [4].

Results of phase II trials with BGB324 have recently been reported. 26 patients with AML received BGB324 in combination with cytarabine or decitabine, and 20 were evaluated for response. Four out of nine patients receiving BGB324 plus cytarabine achieved a complete response with an incomplete hematologic recovery, and two others achieved stable disease. Of the 11 patients receiving decitabine plus BGB324, four achieved a complete response with an incomplete hematologic recovery, and one progressed to stable disease [99]. Another clinical trial evaluated BGB324 in combination with an anti-PD1 immunotherapy in patients with advanced NSCLC. Out of 29 patients, seven partial responses were reported, and 40% of patients with AXL-positive biopsies achieved objective responses. The median progression-free survival for patients expressing AXL was 5.9 months, compared to 4.0 months for AXL negative patients [100]. Clinical trials for BGB324 in TNBC and other cancers are ongoing [101].

#### 14.1.2. SLC-391/SLC-0211

SLC-391/SLC-0211 is a relatively selective small molecule inhibitor of AXL that inhibited the growth of murine colon cancer tumors, likely through stimulation of an anti-immune response [102]. SLC-391/SLC-0211 has also been shown to be effective against AML cells that express high levels of AXL/GAS6 [103]. A clinical trial for safety profiling of SLC-391/SLC-0211 in patients with advanced solid tumors has been established but is not yet recruiting.

#### 14.1.3. TP-0903

TP-0903 showed effectiveness in preclinical models of refractory CLL, neuroblastoma, AML, and other solid cancers, including those that are refractory to other treatments [104–109]. TP-0903 is currently in clinical trials for both advanced stage solid tumors as well as for patients with CLL and small lymphocytic lymphoma.

### 14.2. Dual AXL/MER Inhibitors

#### 14.2.1. INCB081776

This small molecule inhibitor targets both AXL and MER. In xenografts of NSCLC, INCB081776 was shown to inhibit tumor growth in immunocompetent but not in immunocompromised mice, indicating that the effects of this compound could be mediated through an anti-tumor immune response [110]. INCB081776 is currently in an early-phase clinical trial for patients with advanced solid tumors.

#### 14.2.2. ONO-7475

This small molecular inhibitor targets both AXL and MER and is effective against preclinical models of AML [111]. It is currently in clinical trials for acute leukemia.

### 14.3. Others

Several other inhibitors have been developed that are not specific for AXL nor are dual AXL and MER inhibitors. BMS777-607 is a tyrosine kinase inhibitor that blocks the activity of all of the TAM family members. In a murine TNBC model, BMS-777607 in combination with an immunotherapy (anti-PD-1) decreased tumor growth and lung metastases [112]. Additionally, several new AXL inhibitors have been developed, and many have shown promise in preclinical testing. The AXL inhibitor SGI7079 reduced the growth of established NSCLC xenografts and was able to restore sensitivity to EGFR inhibitors in erlotinib-resistant cell lines [80]. Mollard et al. designed a series of AXL kinase inhibitors that inhibited the growth of pancreatic cancer cell lines [113]. Newly developed compound 8i is selective for AXL compared to most other kinases except for FLT3, and it inhibited invasion and migration of MDA-MB-231 cells induced with TGF-*β*, which increases expression of EMT markers [114]. RU-301 and RU-302 are newly developed small molecular inhibitors of the TAM family of receptors by preventing GAS6 binding [115].

### 14.4. Other Considerations

Despite clinical promise, potential problems could arise with any targeted therapy. A recent study showed a positive feedback loop was present in MDA-MB-231 cells whereby treatment with BMS777607 resulted in increased levels of AXL at the cell surface due to inhibition of ubiquitin-mediated lysosomal degradation [26]. This could decrease the effectiveness of this therapy. Signaling through MERTK has also been shown to mediate resistance to AXL-targeted therapies in TNBC preclinical models [116], and activation of HER3 has been implicated in resistance to AXL inhibitors in MDA-MB-231 cells [117]. Therefore, future studies should investigate AXL inhibitors in combination with other targeted therapies or chemotherapeutics in order to be effective in the clinic. Decreased AXL transcript levels in lapatinib-resistant HER2^+^ cells have been observed in response to treatment with estrogen blockers, suggesting clinical potential of AXL inhibitors in combination with estrogen receptor blockers such as fulvestrant or tamoxifen, or aromatase inhibitors, such as letrozole, for instance [37].

## 15. Antibody-Based Therapies

Due to the high level of AXL expression on many tumor types, antibody-based therapies which specifically target AXL could be beneficial in the clinic.

### 15.1. Antibody-Drug Conjugates

#### 15.1.1. BA3011/CAB-AXL-ADC

BA3011 is a conditionally active biologic (CAB) antibody-drug conjugate (ADC) that has shown efficacy in inhibiting the growth of lung, prostate, and pancreatic xenografts [118]. It has entered clinical trials for advanced solid cancers [119].

#### 15.1.2. HuMax-AXL-ADC

HuMax-AXL-ADC is an ADC that due to the antibody portion specifically targets AXL-expressing cells and exposes them to the microtubule-disrupting agent monomethyl auristatin E (MMAE) [120]. In a lung tumor xenograft model complete regression was observed after a single dose, and HuMax-AXL-ADC was also effective against patient-derived xenografts [120]. In preclinical testing HuMax-AXL-ADC has shown efficacy against EGFR-inhibitor-resistant NSCLC [121], as well as melanoma, including multidrug-resistant melanoma [122, 123]. This agent has entered clinical trials to establish the safety profile of HuMax-AXL-ADC in patients with various solid tumors (not including breast) [124].

### 15.2. Monoclonal Antibodies

An early phase 1 clinical trial of BGB149, a function blocking antibody directed against AXL, is currently recruiting (NCT03795142). In preclinical development, treatment of murine breast cancer xenografts with a different monoclonal antibody that blocks GAS6 binding, YW327.6S2, that binds both human and murine AXL, decreased tumor growth, inhibited the activity of tumor-associated macrophages, and decreased metastasis formation from MDA-MB-231 xenografts [125]. The anti-AXL monoclonal antibody 20G7-D9 has also been shown to be effective in management of TNBC breast cancer xenografts and patient-derived xenografts, where it blocks signaling, prevents EMT, reduces tumor growth, decreases migration and invasion, and also decreases metastasis formation [68, 126]. Other mAbs are being investigated as well. MAb173 inhibited the invasiveness of Kaposi sarcoma cells and decreased tumor formation and increased apoptosis in Kaposi sarcoma xenografts [127]. DAXL-88 decreased AXL signaling and migration of ovarian and lung cancer cell lines [128].

### 15.3. Anti-AXL Fc Fusion Protein

AVB-S6-500 is a fusion protein composed of the extracellular ligand-binding domain of the AXL receptor fused to the immunoglobulin G1 Fc domain. As such, this fusion protein binds to GAS6 and prevents its binding to AXL. AVB-S6-500 recently gained “fast-track” status for the treatment of patients with recurrent ovarian cancer, indicating that review of the drug will be expedited by the Food and Drug Administration due to its potential ability to fulfill an unmet life-threatening clinical need. In a mouse model of ovarian cancer treatment with AVB-S6-500 in combination with chemotherapy resulted in smaller tumors than those treated with chemotherapy alone [129]. AVB-S6-500 can also synergize with carboplatin and paclitaxel to reduce ovarian cancer cell growth [130].

Other AXL decoy receptors have been developed, including MYD1-72, which has a high affinity for GAS6 and therefore blocks the interaction between AXL and GAS6. This drug blunts tumor growth and metastasis of mouse mammary tumor cells [131]. The ligand-independent methods of activating in AXL discussed above however indicate that such ligand-blocking therapies could be of limited value since AXL is able to be activated through dimerization with itself and with other receptors, independent of ligand binding. Alternative therapeutic strategies, such as using agents which decrease receptor abundance or block the AXL kinase domain, might therefore be more effective.

### 15.4. CAR T-Based Therapy

CAR T (chimeric antigen receptor-modulated T lymphocyte) therapy targeting AXL was effective in a TNBC preclinical model [132]. Currently, a clinical trial is underway of CCT301-38 for patients with AXL-expressing metastatic renal cell carcinoma upon biopsy.

## 16. Conclusions

Thus far, research into AXL in breast cancer has clearly demonstrated an oncogenic role for this receptor in various breast cancer subtypes, as it is upregulated in breast cancer tumors and can promote tumor formation, EMT, metastasis, and chemoresistance through stimulation of an abundance of intracellular signaling pathways. However, a few key questions about AXL remain to be elucidated. As of yet, no activating mutations have been identified in AXL; thus, mutational status of the receptor cannot serve as a biomarker for therapeutic response. Identification of patient subsets that are likely to benefit from therapeutic inhibition of AXL will be crucial if the above-mentioned AXL inhibitors are to be successful in the clinic. It remains to be seen what the biomarker will be for AXL, whether it will be AXL expression levels, activation status, and/or presence of GAS6 or sAXL. Even once a biomarker has been identified through cell culture work, it will be necessary to determine if this marker can be effectively measured in clinical samples, and accurate analytical tools and perhaps more specific phospho-AXL-directed antibodies may need to be developed. The role of AXL in breast cancer is highly context-dependent, and therefore, it is possible that different biomarkers may be necessary for different breast cancer subtypes. For example, AXL has been shown to interact with HER2 in HER2^+^ breast cancer, but this receptor is not overexpressed in TNBC [4].

As discussed above, AXL has been shown to promote a variety of downstream signaling pathways to mediate its various roles in cancer progression, again, many of which are highly context-dependent. It will be important to further elucidate which downstream pathways are responsible for various roles of AXL in order to better predict potential partnering agents with AXL inhibitors. The role of AXL in chemoresistance further suggests that a treatment approach inhibiting AXL in combination with inhibitors of other molecular targets could be an effective therapeutic strategy for breast cancer treatment. The above-discussed role of AXL in the immune system could suggest that AXL inhibition in concert with one of the newer immune checkpoint blocking therapies could be effective.

Finally, while AXL has been the main TAM family member that has been investigated in breast cancer, future work should also consider the role of other TAM family members, especially given the demonstrated cross-talk between these receptors and the recent finding that protein S can stimulate AXL [9]. If other TAM family members are shown to be involved in breast cancer progression, it could indicate that more broad-spectrum TAM inhibitors as opposed to more highly selective AXL inhibitors may be more effective in breast cancer treatment.

In sum, future work should seek to further uncover AXL's role in the breast tumor so that combination therapies could be developed that co-target AXL along with other intracellular players. AXL inhibitors are beginning to show efficacy in the clinic, and a deeper understanding of AXL's role in breast cancer could lead to a better identification of subsets of patients with potential to benefit from AXL-targeted therapy.

## Figures and Tables

**Table 1:** AXL-targeted therapies currently in clinical trials in the U.S.

Therapeutic	Clinical trial	Phase	Disease
Small molecule inhibitor			
AXL selective			
BGB324/R428/bemcentinib	NCT02424617	Phase 1/2	NSCLC
	NCT02488408	Phase 1/2	Acute myeloid leukemia, myelodysplastic syndrome
	NCT02872259 (active)	Phase 1/2	Melanoma
	NCT02922777 (active)	Phase 1	NSCLC
	NCT03184558 (completed)	Phase 2	^∗^TNBC, inflammatory BC
	NCT03184571 (active)	Phase 2	NSCLC, lung adenocarcinoma, metastatic lung cancer
	NCT03649321 (active)	Phase 1/2	Pancreatic
	NCT03654833 (active)	Phase 2	Mesothelioma
	NCT03824080 (active)	Phase 2	Acute myeloid leukemia, myelodysplastic syndrome
	NCT03965494	Early phase 1	Glioblastoma
SLC-391/SLC 0211	NCT03990454	Phase 1	Solid tumors
TP-0903	NCT02729298 (active)	Phase 1	Solid tumors
	NCT03572634 (active)	Phase 1/2	Chronic lymphocytic leukemia, small lymphocytic lymphoma
Dual AXL/MER			
INCB081776	NCT03522142 (active)	Phase 1	Solid tumors
ONO-7475	NCT03176277 (active)	Phase 1	Acute leukemia
Antibody-based			
Antibody-drug conjugate			
BA3011/CAB-AXL-ADC	NCT03425279 (active)	Phase 1/2	Solid tumors
Enapotamab vedotin/HuMax-AXL-ADC/AXL-107-MMAE	NCT02988817 (active)	Phase 1/2	Solid tumors
Monoclonal antibody			
BGB149	NCT03795142 (active)	Early phase 1	Healthy volunteers
Anti-AXL Fc fusion protein			
AVB-S6-500	NCT03607955	Phase 1	Ovarian, fallopian, peritoneal
	NCT03639246 (active)	Phase 1/2	Ovarian
	NCT04004442	Phase 2	Urothelial carcinoma
	NCT04019288	Phase 1/2	Ovarian, fallopian, peritoneal
	NCT04042623	Phase 2	IgA nephropathy
CAR T-based therapy			
CCT301-38	NCT03393936 (active)	Phase 1/2	Renal cell carcinoma
